# The Lund University Checklist for Incipient Exhaustion–a cross–sectional comparison of a new instrument with similar contemporary tools

**DOI:** 10.1186/s12889-016-3001-5

**Published:** 2016-04-21

**Authors:** Roger Persson, Kai Österberg, Njördur Viborg, Peter Jönsson, Artur Tenenbaum

**Affiliations:** Department of Psychology, Lund University, SE-22100 Lund, Sweden; Department of Laboratory Medicine, Division of Occupational and Environmental Medicine, Lund University, SE-22185 Lund, Sweden; School of Education and Environment, Centre for Psychology, Kristianstad University, SE-29188 Kristianstad, Sweden; Hälsan & Arbetslivet, Occupational Health Care Unit, Region Västra Götaland, Skaraborg Hospital, SE-54185 Skövde, Sweden

**Keywords:** Burnout, Exhaustion disorder, KEDS, LUCIE, Personality traits, s-ED, Stress

## Abstract

**Background:**

Stress-related health problems (e.g., work-related exhaustion) are a societal concern in many postindustrial countries. Experience suggests that early detection and intervention are crucial in preventing long-term negative consequences. In the present study, we benchmark a new tool for early identification of work-related exhaustion–the Lund University Checklist for Incipient Exhaustion (LUCIE)–against other contextually relevant inventories and two contemporary Swedish screening scales.

**Methods:**

A cross-sectional population sample (*n =* 1355) completed: LUCIE, Karolinska Exhaustion Disorder Scale (KEDS), Self-reported Exhaustion Disorder Scale (s-ED), Shirom-Melamed Burnout Questionnaire (SMBQ), Utrecht Work Engagement Scale (UWES-9), Job Content Questionnaire (JCQ), Big Five Inventory (BFI), and items concerning work-family interference and stress in private life.

**Results:**

Increasing signs of exhaustion on LUCIE were positively associated with signs of exhaustion on KEDS and s-ED. The prevalence rates were 13.4, 13.8 and 7.8 %, respectively (3.8 % were identified by all three instruments). Increasing signs of exhaustion on LUCIE were also positively associated with reports of burnout, job demands, stress in private life, family-to-work interference and neuroticism as well as negatively associated with reports of job control, job support and work engagement.

**Conclusions:**

LUCIE, which is intended to detect pre-stages of ED, exhibits logical and coherent positive relations with KEDS and s-ED as well as other conceptually similar inventories. The results suggest that LUCIE has the potential to detect mild states of exhaustion (possibly representing pre-stages to ED) that if not brought to the attention of the healthcare system and treated, may develop in to ED. The prospective validity remains to be evaluated.

**Electronic supplementary material:**

The online version of this article (doi:10.1186/s12889-016-3001-5) contains supplementary material, which is available to authorized users.

## Background

Stress-related health problems are a source of concern in many postindustrial countries, including Sweden, as chronic stress contributes to sickness absence and reduced work performance [1–3]. To facilitate clinical management of individuals with suspected chronic stress, in 2005 The Swedish National Board of Health and Welfare (NBHW) adopted the diagnosis *exhaustion disorder* (ED) (F43.8A) as a further specification within the ICD-10 code F43.8 “Other reactions to severe stress” [4, 5]. In contrast to the related construct *burnout*, which emphasizes the psychological manifestations of exhaustion and facets such as cynicism and professional efficacy [6], ED is primarily characterized by exhaustion, reduced activity level, an increased need for recovery and diverse symptoms (e.g., pain, impaired memory, insomnia) that cause distress in social and/or work life [5]. ED is also differentiated from other conditions that similarly may lead to excessive fatigue, tiredness and exhaustion (e.g., diabetes, heart disease, thyroid disease, substance abuse etc.) as well as adjacent psychiatric diagnoses (e.g., depression, general anxiety, neurasthenia, and dysthymia). In cases where a psychiatric disorder is identified alongside ED, the NBHW recommends that the psychiatric diagnosis be the primary one and ED used only as an additional specification [5] (Table 1). Consistent with theories of stress that center on homeostasis (or allostasis) [7–13], it is assumed that insufficient rest and recovery as well as very frequent, or excessively long lasting, stress responses will lead to an unhealthful physiological imbalance that may lead to ED [5].

**Table 1:** The criteria for exhaustion disorder as proposed by Swedish National Board of Health and Welfare (NBHW,2003)

A.	Physical and mental symptoms of exhaustion with a duration of at least 2 weeks. The symptoms have developed in response to one or more identifiable stressors, which have been present for at least 6 months.
B.	Markedly reduced mental energy is a predominant feature, as manifested by reduced initiative, lack of stamina or increase in time needed for recovery after mental efforts.
C.	At least four of the following symptoms have been present nearly every day, during the same 2-week period:
1.	Concentration difficulties or impaired memory
2.	Markedly reduced capacity to deal with demands or to work under time pressure
3.	Emotional instability or irritability
4.	Sleep disturbances
5.	Marked physical weakness or fatigability
6.	Physical symptoms such as muscular pain, chest pain, palpitations, gastrointestinal problems, vertigo or hypersensitivity to sounds
D.	The symptoms cause clinically significant distress or impairment in social, occupational or other important areas.
E.	The symptoms are not due to the direct physiological effects of any substance (e.g., a drug of abuse, a medication) or a physical illness/injury (e.g., hypothyroidism, diabetes, infectious disease).
F.	If the criteria for major depression, dysthymia or generalized anxiety disorder are met simultaneously, exhaustion disorder is set only as an additional specification to any such diagnosis.

Rehabilitation from ED may require long periods of sick leave (up to 1 year and some individuals may never fully regain their previous level of functioning) and entail complicated rehabilitation procedures [5]. However, the current treatment recommendations given by the NBHW are sparse and lacking in specificity. On the whole (our translation): “*The treatment is based on psychological support intended to create a balance between activity and rest. Symptomatic treatment focuses on supporting sleep and reducing anxiety. Rehabilitation includes lifestyle change, stress management and the gradual return to a normalized life*.”[14]. Although some individuals might develop ED more easily due to variations in biological constitution and/or low access to personal or social resources, ED may in theory affect anyone if the exchange between the individual and the environment is sufficiently challenging, long, and intense. However, a recognized problem when managing ED in, for example, primary health care or occupational health services is the lack of procedures and routines, a situation described as triggering frustration and irritation among healthcare staff [15]. In Sweden, the Self-reported Exhaustion Disorder Questionnaire (s-ED) [16] and the Karolinska Exhaustion Disorder Scale (KEDS) [17] have been developed to facilitate the diagnostic procedure. At present, however, both lack of knowledge concerning the very early indications of ED and lack of specific instruments for detecting such pre-stages prevent healthcare staff from informed decisions about suitable early interventions aimed at reversing or alleviating emerging ED. Yet clinical experience indicates that, at an early stage, rather simple interventions may suffice to reverse development of ED [18]. This insight, and the need for user-friendly and reliable instruments that can assist healthcare personnel in detecting pre-stages of ED, led to the development of the Lund University Checklist for Incipient Exhaustion (LUCIE). LUCIE is a data-driven and empirically grounded inventory that aims to detect the often diffuse pre-stages of ED that may be found in the working population. Building on patient narratives, interviews concerning prodromal stages of ED, systematic analyses of patient medical file data and extant questionnaires, 28 items (across 6 domains) were compiled and adjusted so as to achieve correspondence with patient narratives and experiences regarding onset of ED. All changes in emotions and behaviors that the patients reported as precursors to their subsequent ED were recorded and the most frequently reported changes were used as a basis for creating the LUCIE items (see Additional file 1 for in-depth information on the development of LUCIE).

The present study is part of a longitudinal study involving 11 consecutive surveys made over a 3-year period. Here, we report the cross-sectional baseline benchmarking of LUCIE against the s-ED and KEDS screening scales. Our aims were fourfold: 1) to examine and estimate the degree of agreement between the three screening scales; 2) to examine to what extent LUCIE classifications were associated with reporting in other well-known inventories aimed at assessing ED-related concepts, such as burnout (the Shirom Melamed Burnout Questionnaire (SMBQ) [19]) and work engagement (the Utrecht work engagement scale (UWES) [20]); 3) to examine to what extent LUCIE classifications were related to the reporting of Job demands, Job control and Job support (the Job Content Questionnaire (JCQ) [21]) as well as private life stressors including Family-to-work interference [22]; 4) to examine how personality factors assessed using the Big Five Personality Trait Inventory (BFI) [23] were associated with LUCIE classifications.

## Methods

### Study design

The present cross-sectional observational study is the first (baseline) assessment of a 3-year longitudinal cohort study including 11 equally spaced (i.e., 3 months) consecutive surveys between 2012 and 2014. All data in the present paper were collected using paper-and-pencil questionnaires that were returned by mail. The study protocol was approved by the Regional Ethical Review Board in Lund, Sweden (reg. no. 2012/298). All participants gave written informed consent when they entered the study.

### Participants

A total of 1355 occupationally active individuals (57 % women), with a mean age of 41.1 years (SD 6.7 years; range 27 − 52), were included. At baseline, 54 % had at least three years of university studies, 13 % shorter university studies, 16 % upper secondary school, 16 % lower secondary school, and less than 1 % reported elementary school. The vast majority were employees (90 %), 6 % were self-employed, and 4 % combined self-employment with outside employment. Occupational activity on at least a full-time basis (40 h/week) was reported by 83 %, while 16 % worked 75 − 99 % of full time, and 1 % worked 50 − 75 % of full time (occupational activity from employment < 70 % of full time was accepted in case additional work as self-employed was reported; *n =* 4). A marginal group (3 %) temporarily worked less than full time owing to parallel activities, such as studies or parental leave, or to partial unemployment.

#### Identification of participants

**Fig. 1:**
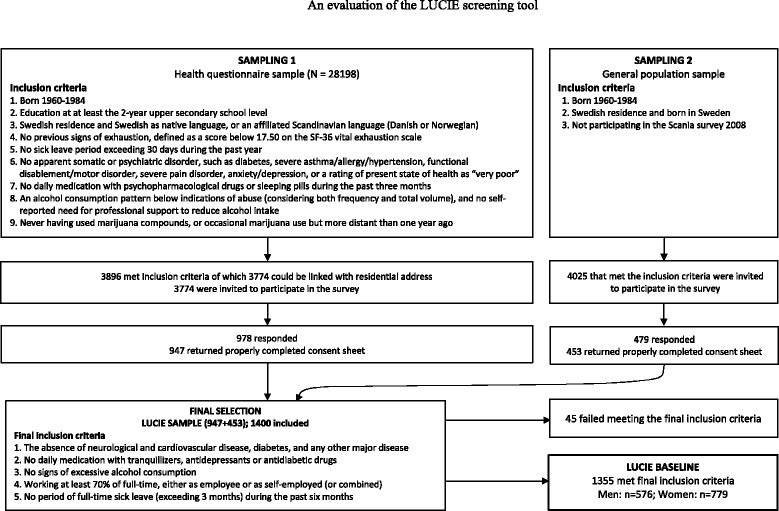
Flowchart of the recruitment procedure

Participants were selected in two sampling rounds (Fig. 1) entailing a pool of responders to a health questionnaire and a supplementary sample from the general population. The target was to obtain circa 1500 individuals who had a relatively long education, were likely to have worked several years in their profession, but who were not approaching retirement. In addition, participants should be in good health, thus without previous somatic or psychiatric disorder, as indicated in their self-reports.

##### Sampling from the health questionnaire

Given our intention to identify the earliest signs of ED, and based on our previous clinical experience of ED cases, we strove to include employees in active work who had a relatively long educational background, assuming such individuals would be engaged in complex and demanding work. This approach was judged to increase the likelihood of prospectively finding individuals with accumulated and prolonged stress reactions that may lead to ED. Access was granted to a database consisting of 28,198 responders to a population survey of lifestyle and health in southern Sweden conducted in 2008 [24]. The inclusion criteria were: 27 − 52 years of age, at least a 2-year upper secondary school education, a Scandinavian language as native language, and no apparent somatic or psychiatric disorder or drug abuse. An invitation letter and a baseline questionnaire were sent to the 3774 eligible individuals, asking them to reply to a similar questionnaire every three months during a 3-year period. The invitation letter requested that participants be gainfully employed (at least 75 % of full time), had no period of long-term full-time sick leave for the past six months, had no chronic disease or prescribed daily medication. In total, 947 individuals returned the baseline questionnaire with the informed consent sheet and were thus included as potential participants.

##### Sampling from the general population

Additional individuals were invited who were on the Scania University Hospital population register, which covers all inhabitants in the southernmost county of Sweden, *Scania (Skåne)*. The inclusion criteria were: 27 − 52 years of age, Swedish residence and born in Sweden, and not already identified in the above health questionnaire sample (information relevant to the other selection criteria were not available for this sample). Thereafter, a randomized sample of citizens living in the major southern Swedish cities of Lund and Malmo, and their vicinity, was drawn. Given our intention to obtain circa 500 additional participants, and an expected participation rate of 10 − 15 %, 4025 eligible individuals were mailed the invitation letter and the baseline questionnaire. In total, 453 individuals returned the baseline questionnaire with the informed consent sheet, and were thus included as potential participants.

##### Final criteria for inclusion

The 1400 returned baseline questionnaires with properly filled in consent sheets were subjected to further scrutiny. Individuals who had a somatic disease, daily medication with psychotropic drugs, excessive alcohol intake, who worked only part time, or had recently been prescribed a longer full-time sick leave were excluded (*n =* 45). Thus the final study sample comprised 1355 individuals.

### Measures

#### Inventories for screening exhaustion disorder (ED)

##### Lund University Checklist for Incipient Exhaustion (LUCIE)

The development of LUCIE is described in Additional file 1 and the questionnaire sheet in Additional file 2. In short, LUCIE consists of 28 items (statements) covering 6 domains: (a) *sleep and recovery* (3 items), (b) *separation between work and spare time* (4 items), (c) *sense of community and support in the workplace* (2 items), (d) *managing work duties and personal capabilities* (5 items), (e) *private life and spare time activities* (3 items), and (f) *health complaints* (11 items). Responses to all items were made on a 4-point scale: 1 = not at all, 2 = somewhat, 3 = quite a bit, 4 = very much. The distinctiveness of the dimensions was verified using a principal component analysis (Additional file 1). Cronbach’s alpha ranged from 0.67 to 0.87 on the domain subscales, and was 0.92 on the full scale (*n =* 1355).

The LUCIE items were derived from qualitative analyses of patient interviews and patient narratives concerning the prodromal stages of ED as well as through systematic analyses of the patients’ journal data (*n =* 92). The scoring builds on two algorithms that generate two supplementary indicators: the *Stress Warning Scale* (SWS) and the *Exhaustion Warning Scale* (EWS). Each scale ranges from 0 to 100 (for a detailed description on the calculation of scale scores see Additional file 1). A SWS score ≤ 17.00 (‘*the green zone’*) is intended to indicate no or negligible lasting stress symptoms. A SWS score between 17.01 and 38.50 (‘*the yellow zone’*) suggests possible slight lasting stress symptoms. A SWS score ≥ 38.51 (‘*the red zone’*) indicates mild to moderate lasting stress symptoms. If the SWS score reaches the red zone, it is advisable to also check the supplementary EWS score, which covers more serious symptoms of lasting stress, thus suggesting exhaustion. An EWS score ≤ 21.50 (‘*the EWS green zone’*) indicates that signs of exhaustion are absent or negligible, while a score exceeding 21.50 (‘*the EWS red zone’*) suggests severe lasting stress symptoms that might indicate ED. In practice, the combination of SWS and EWS scores provides a four-step ranking of incremental stress symptomatology:

1. *Step 1-GG* (SWS green zone and EWS green zone) = no or negligible lasting stress symptoms
2. *Step 2-YG* (SWS yellow zone and EWS green zone) = possible slight lasting stress symptoms
3. *Step 3-RG* (SWS red zone and EWS green zone) = mild to moderate lasting stress symptoms, but less severe than ED
4. *Step 4-RR* (SWS red zone and EWS red zone) = lasting stress symptoms of a severity indicating possible ED.

Because the other theoretically plausible combinations of scores (i.e., SWS green zone or SWS yellow zone in combination with EWS red zone score) are extremely rare these four ranking steps serve as a useful simplification. Thus, in cases where a person obtains a high SWS score, the EWS measure will work as sorting mechanism by indicating whether the stress symptomatology reaches an intensity that is also indicative of ED (step 4), or one that is more benign in nature (step 3).

##### Karolinska exhaustion disorder scale (KEDS)

KEDS is a recently developed tool for screening for the presence of ED [17]. It contains 9 items, covering (1) ability to concentrate, (2) memory, (3) physical stamina, (4) mental stamina, (5) recovery, (6) sleep, (7) hypersensitivity to sensory impressions, (8) experience of demands, and (9) irritation and anger. Each item has seven response alternatives, ranging from 0 − 6, where higher values reflect more severe symptoms. Verbal anchors are provided for the scale steps 0, 2, 4 and 6, but not for 1, 3 and 5. The sum of item scores constitutes the outcome, which may vary between 0 to 54. A sum of item scores ≥ 19 is considered to indicate possible ED [17]. Cronbach’s alpha was 0.86.

##### Self-reported exhaustion disorder scale (s-ED)

The s-ED scale consists of 4 items, one of which consists of 6 sub-items. Its aim is to assess exhaustion in compliance with the Swedish National Board of Health and Welfare (NBHW, 2003) criteria for ED (Table 1) [16]. A classification of s-ED requires that the respondent reply “Yes” to the statements A, B and D (see Table 1) as well as confirm the presence of at least four of the six symptoms specified in C. For statement D, an affirmative reply can be graded with the two alternatives “Yes, a little” and “Yes, pronounced,” which defines the severity of the condition in terms of “light/moderate s-ED” or “pronounced s-ED,” respectively.

#### Inventories pertaining to the burnout concept

##### Shirom-melamed burnout questionnaire (SMBQ)

The SMBQ [19] is a psychologically oriented measure that is based on Hobfoll’s Conservation of Resources Theory, but only as regards energy resources [25, 26]. The SMBQ assesses burnout using 22 items in four domains: (a) “Physical Fatigue,” (b) “Cognitive weariness,” (c) “Tension,” and (d) “Listlessness.” Responses to the items were made on a 7-point scale ranging from 1 ‘Never or almost never’ to 7 ‘Always or almost always.’ Five items require reversed scoring. The mean score of each domain was used as the outcome, and the score SMBQ-Global-22 is represented by the mean of all 22 items. An alternative SMBQ-Global-18, excluding the “Tension” domain, as suggested by Lundgren-Nilsson et al., was also explored [27]. Cronbach’s alpha ranged from 0.76 to 0.94 on the domain subscales and on the Global-22 scale and the Global-18 scale, 0.96 and 0.95, respectively.

##### Utrecht work engagement scale (UWES-9)

The UWES-9 [20] was used to assess work engagement, which is theoretically considered the opposite of burnout. Three aspects of work engagement were assessed using 9 items; the three aspects were: vigor, dedication, and absorption. Each aspect was assessed using three items; responses were made on a 7-point scale with labels ranging from “Never” (score 0) to “Always” (score 6). Results are reported as the mean value of each aspect, and as a grand total mean of all items. Cronbach’s alpha ranged from 0.82 to 0.87 on the aspects, and was 0.93 on the total scale.

#### Inventories pertaining to work conditions, private life, and family-to work interference

##### The job content questionnaire (JCQ)

A Swedish 26-item version of the Job Content Questionnaire (JCQ) [21] was used to assess perceived psychological job demands, job control and job support. The 9 psychological job demands items concern conflicting demands, how hard the workers work, and organizational constraints on task completion. The 9 job control items concern possibilities to make decisions about work (subscale: decision authority, 3 items) as well as the required skill level and possibilities to be creative (subscale: skill discretion, 6 items). The 8 job support items differentiate between instrumental and emotional aspects as well as support from leaders (subscale: manager support; 4 items) and from colleagues (subscale: co-worker support; 4 items) [21]. Each item was given as a statement, where the subject’s response indicated degree of agreement. Responses to the psychological job demands and job control items were made on a 4-point scale (1–4): 1 = totally disagree, 2 = disagree, 3 = agree and 4 = totally agree. Responses to the support items were made on a 5-point scale (0–4): 0 = missing supervisor/colleagues, 1 = totally disagree, 2 = disagree, 3 = agree and 4 = totally agree. For respondents indicating “missing supervisor/colleagues,” support scores were not calculated. Cronbach’s alpha ranged from 0.78 to 0.79 for the main categories job demands, job control and job support, while the subscale alpha values varied between 0.61 and 0.83.

##### Circumstances in private life

*Private stress* One item was created to estimate to what extent factors and circumstances outside work burdened the respondents. The question read “*Would you agree that you have recently felt stressed or mentally strained due to problems or demands outside work?*” and responses were made on a 4-point scale: 1 = Not at all, 2 = To some extent, 3 = Quite a lot, and 4 = Not applicable. The ‘not applicable’ option was recoded to “1” prior to analyses. Thus the score ranged from 1 to 3, with higher scores indicating more stress.

*Family to work interference* This was measured using four items [22]. Two items covered time-based interference, and the other two covered strain-based interference. The unidirectional assessment only considered the influence of the family on work [28]. Responses were made on a 4-point scale indicating agreement: 0 = not at all, 1 = to some degree, 2 = to a large degree, 3 = and does not apply. The “does not apply” option was recoded to zero, and the sum of scores on the four items was used as a family-to-work interference index (range 0-8). Higher scores indicated greater interference. Cronbach’s alpha was 0.67.

#### Inventory pertaining to the concept of personality traits

##### The big five inventory (BFI)

The Big Five personality dimensions–Neuroticism (N), Extraversion (E), Openness (O), Agreeableness (A), and Conscientiousness (C) [29]–were measured using the 44-item BFI [23]. The items of the BFI are short and easily understandable phrases, and each BFI item is rated on a 5-point scale indicating degree of agreement: 1 = “Disagree strongly,” 2 = “Disagree a little,” 3 = “Neither agree nor disagree,” 4 = “Agree a little” and 5 = “Agree strongly.” For each Big Five dimension, the mean score of the 8 to 10 items covering the specific dimension was used as an outcome measure. Cronbach’s alpha for the dimensions were: *N =* 0.79; E = 0.86; O = 0.79; A = 0.73; C = 0.75.

### Statistical analysis

The statistical analysis was performed using the IBM SPSS 22.0 software. Two-tailed *p*-values ≤ 0.05 were considered statistically significant. Kappa statistics and Spearman rank correlations (both with bootstrap estimated 95 % confidence intervals (95 % CI) in order to compensate for ties) were used to estimate the degree of agreement and association between LUCIE, KEDS, and s-ED. For the Kappa statistics, we also conducted sensitivity analyses (i.e., tested the robustness of our result by examining narrower subsets of the study sample by excluding observations that, from a theoretical perspective, may be considered different). Accordingly, and because (a) LUCIE is intended to assess early stages of exhaustion and (b) to estimate agreement between the categories indicating more severe signs of exhaustion, we also conducted analyses in which individuals classified as belonging to the middle steps of the LUCIE 4-step ranking were excluded. For continuous scale scores, non-parametric Kruskal-Wallis tests were used to evaluate whether groups of participants defined by their LUCIE classification differed with regard to their reporting in the other inventories. For nominal or category data, between-group comparisons were made using Pearson’s Chi-square test.

## Results

### Associations between the exhaustion screening inventories and age and gender

Descriptive data on age and gender are presented in Table 2. There was no difference in mean age across the four LUCIE ranking steps (*H* = 3.86, *df* = 3; *p* = 0.277) or the three s-ED categories (*H* = 2.88, *df* = 2; *p* = 0.237). However, participants with an indication of ED in KEDS were slightly younger than participants with no such indication (*H* = 4.19, *df* = 1; *p* = 0.041). The gender distributions across the four LUCIE ranking steps were not significantly different (*χ*^2^ = 4.1, *df* = 3; *p* = 0.26). Proportionally, more women than men fulfilled the exhaustion criteria for KEDS (*χ*^2^ = 10.7, *df* = 1; *p* = 0.001) and s-ED (*χ*^2^ = 12.4, *df* = 2; *p* = 0.002).

**Table 2:** Distribution of men and women across the different exhaustion measures

	Age		Total		Women		Men		Chi-square test
			(*n =* 1355)		(*n =* 779)		(*n =* 576)		
	Mean	SD	N	%	N	%	N	%	*P*-value
LUCIE									0.264
Step 1-GG (*n =* 881)	41	7	881	65	495_a_	64	386_a_	67	
Step 2-YG (*n =* 282)	40	7	282	21	163_a_	21	119_a_	21	
Step 3-RG (*n =* 117)	41	7	117	9	72_a_	9	45_a_	8	
^1^Step 4-RR (*n =* 67)	41	7	67	5	45_a_	6	22_a_	4	
KEDS									0.001
Normal (*n =* 1168)	41	7	1168	87	651_a_	84	517_b_	90	
Exhaustion (*n =* 179)	40*	7	179	13	123_a_	16	56_b_	10	
s-ED									0.002
No (*n =* 1250)	41	7	1250	92	702_a_	90	548_b_	95	
Mild (*n =* 71)	42	7	71	5	54_a_	7	17_b_	3	
Pronounced (*n =* 34)	40	7	34	3	23_a_	3	11_a_	2	

Note: Values in the same row and subtable not sharing the same subscript are significantly different at *p <* 0.05 in the two-tailed test of equality for column proportions

*Kruskal-Wallis test, *p <* 0.05

^1^The rare combination of SWS yellow + UWS red (*n =* 4) was included in this category

### Associations and agreement between the exhaustion screening inventories

Descriptive data on the prevalence of exhaustion signs are presented in Table 2, showing that the prevalence rates were 13.4, 13.8 and 7.8 % on the LUCIE, KEDS and s-ED, respectively. Table 3 shows the overlap between LUCIE, KEDS and s-ED.

**Table 3:** Descriptive cross-tabulation of the overlap between LUCIE and KEDS or s-ED

LUCIE										
KEDS	Step 1-GG		Step 2-YG		Step 3-RG		^1^Step 4-RR		Total	
	N	%	N	%	N	%	N	%	N	%
Normal	855	97.4	221	79.8	58	49.6	26	38.8	1160	86.6
Exhaustion	23	2.6	56	20.2	59	50.4	41	61.2	179	13.4
Total	878	100	277	100	117	100	67	100	1339	100
LUCIE										
s-ED	Step 1-GG		Step 2-YG		Step 3-RG		^1^Step 4-RR		Total	
	N	%	N	%	N	%	N	%	N	%
No	867	98.4	252	89.4	82	70.1	41	61.2	1242	92.2
Mild	12	1.4	22	7.8	26	22.2	11	16.4	71	5.3
Pronounced	2	0.2	8	2.8	9	7.7	15	22.4	34	2.5
Total	881	100	282	100	117	100	67	100	1347	100

^1^The rare combination of SWS yellow + UWS red (*n =* 4) was included in this category

Spearman rank correlations indicated that all indicator categories were positively associated with each other. LUCIE correlated with s-ED (rho = 0.37, 95 % CI = 0.31-0.42) and KEDS (rho = 0.49, 95 % CI = 0.44-0.54), and s-ED and KEDS were also correlated (rho = 0.48, 95 % CI = 0.40-0.56).

When comparing the kappa agreement between LUCIE, KEDS and s-ED using all indicator categories, kappa for LUCIE and s-ED was 0.13 (95 % CI = 0.09-0.16), and for LUCIE and KEDS 0.21 (95 % CI = 0.17-0.25) (Table 4). The kappa for s-ED and KEDS was 0.35 (95 % CI = 0.29-0.42). In addition, a series of analyses in which the cutoff point for the four LUCIE ranking steps was incrementally alternated showed that the highest agreement was reached when the cutoff point was set between step 2-YG and step 3-RG. The subsequent sensitivity analyses, in which individuals on step 2-YG and 3-RG were excluded, confirmed this pattern, showing a degree of agreement between the more severe LUCIE ranking steps and the other inventories of Kappa = 0.60.

**Table 4:** Estimated agreement between LUCIE and KEDS or s-ED (at different LUCIE cutoff points and when making a sensitivity analysis by excluding identifications in the middle range of LUCIE)

LUCIE^1^		KEDS		s-ED		s-ED	
		(2-levels)		(3-levels)		(2-levels)	
Gliding cutoff point	N	Kappa	[95 % CI]	Kappa	[95 % CI]	Kappa	[95 % CI]
1, 2, 3, 4	1339	0.21	0.17 − 0.25	0.13	0.10 − 0.16	0.12	0.09 − 0.16
1–(2, 3, 4)	1339	0.36	0.32 − 0.42	0.18	0.14 − 0.21	0.22	0.19 − 0.27
1, 2–(3, 4)	1339	0.48	0.41 − 0.55	0.28	0.21 − 0.35	0.36	0.28 − 0.44
1, 2, 3 − (4)	1339	0.28	0.20 − 0.36	0.17	0.10 − 0.24	0.26	0.17 − 0.34
Excluding steps (-)							
1, (2), 3, 4	1062	0.60	0.52 − 0.66	0.32	0.26 − 0.39	0.41	0.33 − 0.49
1, (2), (3), 4	945	0.60	0.49 − 0.70	0.32	0.21 − 0.41	0.46	0.32 − 0.58

Note: Confidence intervals calculated with bootstrap estimation

^1^The rare combination of SWS yellow + UWS red (*n =* 4) was included in LUCIE category-4/RR

When further examining the overlap on the individual level, it was observed that 3.8 % (*n =* 51) of all participants had simultaneous indications of exhaustion on LUCIE (fulfilling the milder ranking step 3-RG criterion), s-ED (including any sign of s-ED), and KEDS. Further, 17.6 % (*n =* 235) of all participants acknowledged exhaustion on one or two inventories. Hence, 21.4 % (*n =* 286) of all participants showed an indication of exhaustion on at least one of the three instruments. Accordingly, of all the 286 participants with an indication of exhaustion on *any* of the instruments, 51 (1/6 of the participants with *any* indication) were identified by *all three* instruments.

### Relationships between LUCIE, SMBQ and UWES

For the SMBQ, significantly higher scores were seen for all subgroups with indications of exhaustion on LUCIE compared to the subgroup without such indication. A trend toward gradually rising SMBQ scores was observed in relation to increasing signs of stress in the LUCIE ranking steps (Table 5).

**Table 5:** Relationships between LUCIE and the SMBQ, UWES, JCQ and BFI scores

	LUCIE								
	Step 1 GG		Step 2 YG		Step 3 RG		^1^Step 4 RR		Kruskal-Wallis ANOVA
	(*n =* 881)		(*N =* 282)		(*N =* 117)		(*N =* 67)		
	Mdn	(10 − 90)	Mdn	(10 − 90)	Mdn	(10 − 90)	Mdn	(10 − 90)	*P*-value
SMBQ									
Global-22	2.1_a_	(1.4 − 3.3)	3.3_b_	(2.2 − 4.5)	4.0_c_	(3.0 − 4.9)	4.6_c_	(3.1 − 5.7)	<0.001
Global-18	2.0_a_	(1.3 − 3.3)	3.2_b_	(2.1 − 4.5)	4.0_c_	(2.9 − 5.1)	4.6_c_	(2.9 − 5.8)	<0.001
Cognitive weariness	1.8_a_	(1.0 − 3.5)	2.8_b_	(1.5 − 4.8)	3.7_c_	(2.0 − 5.3)	4.7_c_	(2.5 − 6.7)	<0.001
Listlessness	2.5_a_	(1.5 − 4.0)	3.5_b_	(2.3 − 5.0)	4.3_c_	(3.0 − 5.3)	4.5_c_	(3.0 − 6.0)	<0.001
Physical exhaustion	2.0_a_	(1.3 − 3.4)	3.3_b_	(2.0 − 4.5)	4.0_c_	(2.9 − 5.4)	4.8_c_	(3.1 − 6.0)	<0.001
Tension	2.3_a_	(1.3 − 3.8)	3.3_b_	(2.0 − 5.0)	4.0_c_	(2.8 − 5.0)	4.5_c_	(3.0 − 6.0)	<0.001
UWES									
UWES Total	4.6_a_	(3.2 − 5.4)	4.2_b_	(2.8 − 5.2)	4.0_c_	(2.4 − 5.2)	3.7_c_	(2.0 − 5.0)	<0.001
Absorption	4.3_a_	(2.7 − 5.7)	4.3_acd_	(2.7 − 5.3)	4.0_b__,c_	(2.0 − 5.3)	4.0_b__,d_	(2.0 − 5.3)	0.003
Dedication	4.7_a_	(3.3 − 6.0)	4.3_b_	(3.0 − 5.5)	4.0_c_	(2.7 − 5.7)	3.7_c_	(1.7 − 5.3)	<0.001
Vigor	4.7_a_	(3.0 − 5.3)	4.0_b_	(2.7 − 5.0)	3.7_c_	(2.0 − 5.0)	3.3_c_	(1.3 − 5.0)	<0.001
JCQ									
Job Control, total	3.3_a_	(2.8 − 3.8)	3.3_b_	(2.7 − 3.8)	3.2_b_	(2.5 − 3.8)	3.2_b_	(2.4 − 3.8)	0.002
-Decision latitude	3.3_a_	(2.7 − 4.0)	3.3_b_	(2.3 − 4.0)	3.0_b_	(2.3 − 3.7)	3.0_b_	(2.0 − 3.7)	<0.001
-Skill discretion	3.3	(2.8 − 3.8)	3.3	(2.7 − 3.8)	3.3	(2.7 − 3.8)	3.5	(2.5 − 3.8)	0.393
Job Demands	2.7_a_	(2.1 − 3.1)	2.9_b_	(2.3 − 3.4)	3.0_c_	(2.6 − 3.7)	3.3_c_	(2.6 − 3.8)	<0.001
Job Support, total	3.0_a_	(2.6 − 3.5)	2.9_b_	(2.3 − 3.4)	2.8_c_	(2.1 − 3.3)	2.7_c_	(1.6 − 3.3)	<0.001
-Sup. Colleagues	3.0_a_	(2.8 − 3.8)	3.0_b_	(2.5 − 3.8)	3.0_c_	(2.5 − 3.5)	3.0_c_	(2.0 − 3.8)	<0.001
-Sup. Manager	3.0_a_	(2.3 − 3.5)	2.7_b_	(1.8 − 3.3)	2.5_c_	(1.5 − 3.5)	2.3_c_	(1.0 − 3.3)	<0.001
BFI									
Neuroticism	2.3_a_	(1.5 − 3.0)	2.5_b_	(1.8 − 3.4)	2.9_c_	(2.0 − 3.6)	2.9_c_	(2.1 − 3.9)	<0.001
Extraversion	3.8_a_	(2.8 − 4.5)	3.5_b_	(2.6 − 4.5)	3.5_b_	(2.8 − 4.3)	3.4_a__,b_	(2.5 − 4.6)	0.019
Openness	3.3_a_	(2.6 − 4.2)	3.4_b_	(2.6 − 4.3)	3.3_a__,b_	(2.7 − 4.1)	3.7_c_	(2.9 − 4.5)	0.001
Agreeableness	4.0_a_	(3.4 − 4.6)	3.9_b_	(3.2 − 4.4)	3.8_b_	(3.2 − 4.4)	4.0_a__,b_	(3.1 − 4.4)	<0.001
Conscientiousness	4.0_a_	(3.3 − 4.6)	3.9_b_	(3.3 − 4.6)	3.9_b_	(3.1 − 4.6)	4.0_a__,b_	(3.1 − 4.7)	<0.001

Note: Values in the same row and subtable not sharing the same subscript are significantly different at *p <* 0.05 in pairwise comparison

^1^The rare combination of SWS yellow + UWS red (*n =* 4) was included in this category

*SMBQ* shirom-melamed burnout questionnaire, *UWES* utrecht work engagement scale, *JCQ* job content questionnaire, *BFI* big five inventory

Data represent the median (Mdn) and the 10^th^ and 90^th^ percentile (10 − 90)

The UWES scores were significantly lower among individuals fulfilling any stress indication on the LUCIE (Table 5). A trend toward a gradual decrease of the UWES global score was observed in relation to increasing signs of stress across the LUCIE ranking steps (*p <* .05 for each step), though not statistically significantly lower for the step 4-RR compared to step 3-RG. Similar trends in LUCIE were observed for each of the three UWES subscales, though they were less pronounced for the UWES Absorption subscale.

#### Relationships between LUCIE and JCQ

There was a clear trend of increasing job demands scores, and decreasing scores for job support, with increasing severity of stress signs across the LUCIE ranking steps (Step 1-GG, Step 2-YG and Step 3-RG/Step 4-RR). Lower job control scores was seen for ranking step 2-YG to 4-RR compared to Step 1-GG (Table 5).

### Relationships between LUCIE and ratings of private stress, interference from family to work life and personality traits

Self-ratings of private stress were observed to be substantially more common among participants with an outcome indicative of slight or pronounced exhaustion on the LUCIE. While only 9 % of participants without any indication on the SWS scale rated “Quite a lot” private stress lately, this rating was two- to fourfold more common among those with any LUCIE ranking step above Step 1-GG, following a ladder of more frequent reports with increasing LUCIE indications of stress/exhaustion (Table 6).

**Table 6:** Relationships between LUCIE and self-rated circumstances in private life and family-to-work interference

	LUCIE								
	Step1 GG		Step 2 YG		Step 3 RG		^1^Step 4 RR		Chi-square test
	(*n =* 881)		(*n =* 282)		(*n =* 117)		(*n =* 67)		
	%	N	%	N	%	N	%	N	*P*-value
Private life									<0.001
Not at all	51_a_	446	31_b_	88	28_b_	33	31_b_	21	
To some extent	41_a_	357	50_b_	139	45_a,b_	53	31_a_	21	
Quite a lot	9_a_	76	19_b_	53	26_b,c_	31	37_c_	5	
									Kruskal-Wallis ANOVA
Family-to-work interference	Mdn	10 − 90	Mdn	10 − 90	Mdn	10 − 90	Mdn	10 − 90	*P*-value
Total sum score^2^	1.0_a_	0 − 4.0	2.0_b_	0 − 5.0	3.0_b_	0 − 6.0	3.0_b_	0 − 6.0	<0.001

Note: Values in the same row and subtable not sharing the same subscript are significantly different at *p <* 0.05 in pairwise comparison

^1^ The rare combination of SWS yellow + UWS red (*n =* 4) was included in this category

^2^ Data represent the median (Mdn) and the 10th and 90th percentile

In addition, the family-to-work interference index was significantly higher among individuals who had any indication of stress signs on LUCIE (ranking steps 2 − 4), compared to those with no LUCIE indication (ranking step 1-GG) (Table 6).

Regarding personality traits, we observed that the BFI neuroticism scores were higher in the subgroups with indications of exhaustion (Table 5). No similar general trend was seen for any other BFI dimension, although scattered differences appeared between subgroups. For example, for individuals with slight to moderate LUCIE stress indication (ranking steps 2 and 3), compared to those without such an indication and ranking step 4, we observed marginally lower scores on the BFI dimension Extraversion. In addition, the subgroup with an exhaustion warning on LUCIE (ranking step 4-RR), reported higher levels of BFI Openness compared with the other subgroups in each scale, respectively.

## Discussion

In the present study including 1355 occupationally active individuals, we compared LUCIE, a new instrument for assessing emerging exhaustion, with two extant Swedish exhaustion screening instruments (i.e., KEDS and s-ED). In addition, we benchmarked LUCIE with other well-known scales related to burnout (SMBQ), work engagement (UWES), work stress (JCQ), private life stressors and personality traits (BFI).

### Principal findings

Increasing signs of exhaustion in LUCIE were positively associated with signs of exhaustion on the KEDS and s-ED inventories, and the associated prevalence rates were 13.4, 13.8 and 7.8 %, respectively. Nonetheless, the overlaps between the inventories on the individual level were at best moderate. Only 51 participants, corresponding to 1/6 of the participants with indications of exhaustion on any inventory, were identified simultaneously by all three instruments. Increasing signs of exhaustion on LUCIE was also positively associated with reports of burnout, job demands, stress in private life, family-to-work interference and neuroticism as well as negatively associated with reports of job control, job support and work engagement.

### Strengths and weaknesses of the study

One strength of the present study was the large and occupationally diverse study sample that was selected to mirror a segment of the working population believed to be at risk for developing ED. The fairly equal distribution of men and women (57 % women) and the fact that the study design allowed for simultaneous evaluation of the relationship between the three exhaustion inventories as well as the relationships between LUCIE and other well-established inventories, are regarded as strengths.

Two important limitations of the current study, however, concern the selection of participants and the cross-sectional design. Regarding the selection of participants, it should first be noted that the response rates were fairly low. Second, it should be noted that, in line with our intentions to identify early signs of ED in a longitudinal study, at baseline we sampled reasonably healthy participants who were working and had a relatively long educational background and work history. This positive selection of participants may have led to a low prevalence of ED. For example, in the initial validation study with the s-ED instrument, in which a random sample of public sector employees were studied, Glise et al. (2010) found a prevalence of 16 % fulfilling the s-ED exhaustion criteria. This is almost twice the prevalence rate observed in the present study sample. However, in contrast to our study in which 57 % were women, the sample obtained by Glise et al. comprised 85 % women. Hence, we cannot exclude the possibility that gender differences in symptom reporting and/or occupational confounding have contributed to the observed differences in prevalence rates in the s-ED inventory. Then again, in the present study neither LUCIE nor s-ED and KEDS were found to be particularly sensitive to variations in gender, although s-ED and KEDS identified a slightly higher proportion of women than LUCIE did.

With a cross-sectional design, it is obviously not advisable to make deductions about causal relationships between the study variables. In addition, and because all of the data were collected with self-report inventories, there is a risk for common method bias (e.g., common rater effects) [30]. If this is the case then this bias is likely to inflate the associations between the variables. On the other hand, the trustworthiness of the results from LUCIE, s-ED and KEDS is somewhat increased by the fact that the participants had to have acknowledged the presence of several symptoms, and at a certain magnitude, in order to be classified as a case. Using multiple indicators for classification, however, does introduce a risk for unintentional content overlap. Indeed, if we uncritically compare the broad LUCIE, KEDS and s-ED constructs with inventories covering narrower constructs that tap their content (e.g., sleep, job demands, fatigue, concentration problems, etc.), we may spuriously inflate associations and end in the triviality trap. It should therefore be emphasized that the present study was not intended to disentangle any potential causal relationship (e.g., high job demands lead to signs of exhaustion in LUCIE or vice versa), but rather to benchmark LUCIE against s-ED, KEDS and other constructs of relevance to ED. Accordingly, content overlap is of lesser concern and in fact part of the study design.

Finally, our use of multiple statistical comparisons also warrants a brief note. Performing many statistical tests seems to call for restricting the number of statistical comparisons or for a downward adjustment of the *p*-value. However, given the descriptive nature of the study, we have chosen to keep a constant 5 % level. Thus, it is advisable to interpret individual significant results with caution.

### Associations and agreement between LUCIE, KEDS and s-ED

The observed low degree of overlap between the three ED instruments may seem disconcerting and to require an explanation. To some extent, the weak agreement verifies the fact that LUCIE focuses on early signs of exhaustion and that the questions are not identical across instruments. In addition, the causes underlying ED may be multifold and complex and evoke different manifestations of symptoms among individuals. Indeed, ED, like related psychiatric diagnoses (cf. major depressive disorder), is a fuzzy syndrome and, accordingly, has no unique set of signature symptoms whose presence indisputably verifies the condition [31, 32]. Because LUCIE, KEDS, and s-ED have been developed in different geographical regions of Sweden (LUCIE, Skåne; s-ED, Västra Götaland; KEDS, Stockholm), we cannot exclude the possibility that associated socio-cultural aspects (e.g., labor market conditions, life-style habits and/or other geographical or regional conditions) have to some extent influenced the manifestations of ED and subsequently the selection of items in the screening instruments. The low agreement may also in part be a statistical artifact resulting from the design of the inventories and modes of classification. The fact that LUCIE operates with four levels, KEDS with two levels, and s-ED with three levels is likely to reduce the kappa values, as the kappa statistic is sensitive to the number of categories included in the analysis [33]. Then again, the instruments are positively associated with each other and the observed overlap between LUCIE and KEDS and s-ED seems to improve with increasing severity of exhaustion signs (cf. Tables 3, 4 and 5). It is possible that the overlap would have been higher if we had used less strict inclusion criteria at baseline and allowed participants with signs of declining health to participate. Thus, in clinical settings, the overlaps between the instruments are probably higher. Nonetheless, and from a practical perspective, the degree of agreement suggests that the three different methods to some extent identify different individuals, particularly in milder/borderline states of exhaustion. Thus, comparisons across methods should be made with caution.

### Associations with ED-related concepts: burnout and work engagement

The analyses between LUCIE and the well-known burnout questionnaire SMBQ [19] and the work engagement questionnaire UWES [20], showed a stepwise increase in all SMBQ scores that paralleled the increase in severity in LUCIE. Individuals fulfilling the criteria for exhaustion reported also significantly lower work engagement on the UWES. Despite the fact that the SMBQ and UWES represent slightly different theoretical approaches, the LUCIE seems to tap into something similar. Thus, it seems plausible that these different self-report methods capture similar, but also partially different, aspects of the underlying neurophysiological and psychological reactions and behavior associated with adaptation to environmental stressors, including stress reactions that originate from the workings of our inner life.

### Associations with perceived circumstances at work and in the private domain

The fact that higher job demand scores on JCQ were incrementally associated with severity of indications on the LUCIE ranking steps suggests that high job demands is an important component of the development toward exhaustion. This observation is in line with a recent review that claimed, with some reservations, to find moderate support for the notion that high job demands can lead to depression [34]. In any event, the perceived work situation is not the only component of interest. Self-reported stress within the private sphere and higher family-to-work interference were also commonly reported by participants showing an indication of ED on LUCIE. This verifies to some extent the notion that stressors within the family and other areas of private life are components of interest in the development toward ED. Even if some studies suggest that workload is a relatively more important factor with regard to developing symptoms than stress in private life is [35, 36], a combination of parallel private and work life stressors is likely to pose the greatest coping challenge. This is in good accordance with our clinical experience of patients diagnosed with ED, who often exhibit some degree of deterioration in both areas. However, it is often difficult to retrospectively disentangle the relative contributions of work stressors and private stressors, not to mention the fact that combined exposure over time may result in even stronger, synergistic effects.

### Associations with personality reporting

Personality dispositions are commonly thought to constitute vulnerability factors or resilience factors in the face of stress. In the present study, our main observation was the stepwise increase in neuroticism scores that paralleled increasing severity of exhaustion on LUCIE. This may be understood in several ways. For example, it is possible that individuals with high neuroticism have a bias toward reporting higher levels of negative affectivity, annoyance and health problems, resulting in a false impression of exhaustion. Indeed, it is known that neuroticism-related traits are associated with reporting of a broad a range of distress symptoms in working populations [37, 38]. Another possibility is that exhaustion-related psychological and physiological changes have already taken place and that the elevated levels of neuroticism reflect signs of exhaustion. It may also briefly be noted that the scores on the Conscientiousness dimension of the BFI showed small variations. It is possible that personality traits narrower than those assessed in the BFI, for example *perfectionism* [39], may be more specifically related to ED.

## Conclusion

The newly developed LUCIE questionnaire, intended to detect pre-stages of ED, showed weak agreement with KEDS and s-ED, two extant Swedish screening tools for detection of exhaustion. Still, LUCIE showed a consistent pattern of relationships to other well-known inventories measuring related constructs such as burnout (SMBQ) and work engagement (UWES) as well as Job demands-control-support, private stress, and family-to-work interference. Accordingly, the results from the present cross-sectional study suggest that LUCIE, which combines a fine-grained analysis of subtle stress symptoms (SWS) with more pronounced symptoms (EWS) into a 4-step ranking of incremental signs of exhaustion, has the potential to detect mild states of exhaustion (possibly representing a pre-stage to ED) that, if not brought to the attention of the healthcare system and treated, may develop into ED. However, the prospective validity of the tool remains to be evaluated.

### Availability of data and materials

Consistent with the study protocol approved by the Regional Ethical Review Board, anonymized data is stored locally at the Division of Occupational and Environmental Medicine, Lund University, Lund, Sweden. Because the participants (in accordance with the approved study protocol) were assured that the crude data should not be published on the internet, access to data will only be granted to eligible researchers wanting to audit our research.

## Additional file

Additional file 1: Initial development of the Lund University Checklist for Incipient Exhaustion (LUCIE) (DOCX 100 kb) Additional file 2: The LUCIE questionnaire, translated from the Swedish original (DOCX 512 kb)
