# Supplementary material for: The Lund University Checklist for Incipient Exhaustion–a cross–sectional comparison of a new instrument with similar contemporary tools
Source: BMC Public Health. 2016 Apr 21;16:350. doi: 10.1186/s12889-016-3001-5 (PMC4839117; doi:10.1186/s12889-016-3001-5)
Supplement: Additional file 1: — Initial development of the Lund University Checklist for Incipient Exhaustion (LUCIE) (DOCX 100 kb) [file 12889_2016_3001_MOESM1_ESM.docx]

**Additional file 1. Initial development of the Lund University Checklist for Incipient Exhaustion (LUCIE)**

**Background and rationale: The need for a measure of early signs**

LUCIE is a data-driven and empirically grounded inventory that aims to assess pre-stages of exhaustion disorder (ED). LUCIE was developed in 2006 as a response to a perceived need for an instrument specifically designed to detect pre-stages of ED. Illness due to work stress had, at that time, rapidly emerged as one of the major causes of health problems and long-term sick leave in Sweden. It was known that when the stress process had been so long lasting that it had led to ED, rehabilitation was often complicated and sick leaves were typically long.

The strong desire to detect ED in its pre-stages, at a time when minor interventions could reverse the development towards ED, was the driving force behind the development of LUCIE. The developers had recently interviewed around 100 patients on sick leave due to ED; these patients had also participated in a workplace intervention project [[1](#_ENREF_1), [2](#_ENREF_2)]. The researchers noticed that most of the research participants (patients) had spontaneously described rather similar and quite conspicuous early signs of what was later to become ED, long before their actual sick leave.

When the development of LUCIE started, assessment of ED entailed various methods. Some methods focused entirely on subjective health complaints, while others used questionnaires that were long and time-consuming, some of which also required psychological expertise for proper interpretation. However, no method or self-rating scale embraced assessment of early signs. Accordingly, building on the research participants’ (patients’) narratives, a number of early changes in behavior and emotional status, which were of significance for the development of ED in its early stages, could be identified.

**The crafting of a new questionnaire**

To cover all of the more commonly reported early signs of ED, it was necessary to create a new measure. Accordingly, a new combination of questionnaire items was compiled. Some items were imported (or modified) from various established questionnaires, for example, the Karolinska Sleep Questionnaire [[3](#_ENREF_3)], Maslach Burnout Inventory – General Survey [[4](#_ENREF_4)] QPS-Nordic [[5](#_ENREF_5)], Lund Subjective Health Complaints [[6](#_ENREF_6)] Work-Family Conflict [[7](#_ENREF_7)] and the Karolinska burnout questionnaire [[8](#_ENREF_8)]. However, other items had to be developed.

Through a qualitative analysis of the self-report data from the medical files of 92 ED patients (and one year’s personal experiences of the same patients), a condensed set of critical signs of incipient ED were combined in a pre-version of LUCIE, which contained 41 items. Six critical themes emerged:

(A) *Sleep and recovery* (4 items) - e.g., problems falling asleep, superficial sleep and early awakening.

(B) *Separation between work and spare time* (5 items) – e.g., tendency to brood over work problems and inability to relax during leisure time.

(C) *Sense of community and support in the workplace* (4 items) - e.g., low quality of social relations and cooperation with peers and/or supervisors

(D) *Managing work duties and personal capabilities* (7 items) - e.g., recurrent feelings of losing control of work tasks/work situation; failing professional efficacy

(E) *Private life and spare time activities* (5 items) - e.g., low energy/interest in social activities with friends/family and waning interest in what were regular leisure activities, hobbies, etc.

(F) *Health complaints* (16 items) - a multitude of bodily and mental complaints associated with long-term stress; e.g., heart palpitations, headache, sensitivity to light and noise, gastrointestinal problems, breathing problems, vertigo or anxiety, memory impairment and irritability.

Within each critical theme A-F, 1 or 2 items that, based on experience, were unrelated to incipient ED (e.g., motor problems, tactile impairment, snoring) were used to control for a generally affirmative response style.

The wording of the questions was intended to be neutral and “non-emotional.” However, the response format encouraged the respondent to disclose the perceived intensity: “To what extent have you felt or noticed....” The response was rated on a 4-point scale: 1= not at all, 2 = somewhat, 3 = quite a bit, and 4 = very much. The aim was to separate between a mainly affirmative response (scale steps 3-4) and a response that indicated negligible intensity (scale steps 1-2). It was presumed that responses on steps 3 and 4 were of relevance, while the lower steps reflected unimportant variation (“noise”) commonly occurring in healthy populations.

**Pilot-testing a pre-version of LUCIE in the target group**

Two strands of pilot testing occurred, one directed to former patients and one to the general population. Thus, and to begin with, a pre-version of LUCIE was mailed to former ED patients (n=91), asking them to look back on the six months preceding their initial sick leave when rating the questions. Second, the pre-version of LUCIE was also given to two groups from a population cohort, that on two previous occasions, at a five year interval, had replied to a regional population health survey (in the southern county of Sweden, Scania). This survey contained the Job Content Questionnaire (JCQ), which is based on the Demand-Control-Support Model [[9-11](#_ENREF_9)], and which had been completed by 6770 occupationally active individuals on both occasions.

For the purpose of obtaining one population group with a presumed elevated risk for acquiring ED and a control group with a presumed low risk, we selected respondents who had a constantly high or low workload on the JCQ. A constantly high workload was defined as having a work situation that, on both test occasions, fulfilled the JCQ criteria for ”Job Strain” (JS; n=295), e.g. high demands and low control. The control group was defined as "No Job Strain" combined with "Not Low Support" (NJS+NLS; n=511, randomized) on both occasions. These subgroups of the population health survey replied to the pre-version of LUCIE; they were asked to consider ”the past month” when rating the questions.

*Prioritizing questions and creating indicators*

A Principal Component Analysis (Varimax with Kaiser Normalization) essentially confirmed the six themes of annoyance (A-F). In addition, high internal consistency was observed for the full LUCIE scale: Cronbach’s alpha was 0.95 in the population cohort and 0.83 in the former ED group. The questions included to control for a generally affirmative response style were acknowledged by extremely few participants, thus indicating good discriminant validity. For the target questions, a clear gradient in the groups’ replies was observed: The former ED group replied in the affirmative to most target questions, followed by less frequent and less high affirmative answers in the JS group and very few affirmative answers in the NJS+NLS group.

In an effort to reduce the number of items so as to facilitate the response process and create maximal separation between the groups, each item was scrutinized in the following way:

(A) Identification of each item where an affirmative answer was substantially more frequent in the *combined* former ED and JS groups compared to the NJS+NLS group.

(B) The affirmative answer was sustained as a possible early sign of exhaustion by an even higher frequency of affirmative answers in the former ED group than in the JS group, and had to include at least 60% of the former ED patients.

The purpose of this selection criterion was to collect a set of items (and scale steps) that could identify milder long-term stress symptomatology, the goal being to detect *pre-stages* of exhaustion.

(B) Identification of each item (and scale step) that was responded to in the affirmative substantially more frequently by the former ED group than by the *combined* JS and NJS+NLS groups.

This selection criterion aimed to identify only more severe states of exhaustion, such as ED.

Detailed criteria for selecting suitable items are shown in Table A1.

Table A1. Criteria used in selecting items for the abbreviated LUCIE scale.

| Purpose | A. Sensitive marker for mild long-term stress symptomatology | B. Restrictive marker for severe long-term stress symptomatology |
| --- | --- | --- |
| Scale  Group | The SWS-scale  = separating (NJS+NLS) from (JS *and* ED) | The EWS-scale  = separating ED from (NJS+NLS *and* JS) |
| Former ED | 1. ≥ 60% of the former ED group replying on scale steps 3+4 (”quite a bit” and ”very much”)  ***and***  2. A higher frequency of replies on scale steps 3+4 in the former ED group than in the  JS group | 1. Scale steps on which former ED patients replied at least **4 times more frequently** than in the JS *and* NJS+NLS groups  ***and***  2. ≥ 60% of the former ED patients replying on scale steps 3-4 |
|  |  | ***or***  3*.* Scale steps on which replies were **at least 8 times more frequent** among former ED patients than in *both* the NJS+NLS *and* JS groups |
| Job Strain | 1. A frequency of replies on scale steps 3+4 at least **twice higher** in the JS group than in the NJS+NLS group | - |

Combining the criteria led to the selection of 28 items according to criterion A, of which 26 items was also selected according to criterion B. Computational algorithms were used to produce separate outcome scales for criterion A and criterion B:

***(A) SWS=the Stress Warning Scale*** aimed at capturing milder long-term stress symptomatology in order to detect *pre-stages* of exhaustion. To construct the SWS scale, item scores for 26 items (excluding item 10 and 13) were dichotomized in the middle of the response scale (between the scale steps “somewhat”[2] and “quite a bit”[3]), which means that a reply on scale step 3 (or 4) was scored as “1” and a reply on scale step 1 or 2 was scored as “0.” The means of the summed scores within each problem area/dimension (A-F) were calculated separately, resulting in six mean values for the six problem areas/dimensions, each with a possible scoring range between 0 and 1. These six mean scores were transformed into a total score by computing the mean of the means, leading to an equal weighting of each problem area/dimension (A-F). For convenience, the scoring range was multiplied by a factor of 100, providing a final SWS score that ranged from 0 to 100. Further analyses, aiming to optimize separation between groups, led to a suggested cutoff score of SWS=38.5, which identified 99% of the former ED patients and 30% of the JS individuals, while only 8% of the NJS+NLS individuals were falsely identified. An alternative cutoff score of SWS=17.0 proved to detect 100% of the ED and 57% of the JS individuals, versus 23% of the NJS+NLS individuals. Thus, this lower cutoff score gave a more sensitive, but probably less specific indication of emerging stress symptomatology.

***(B) EWS=the Exhaustion Warning Scale****,* which was intended to differentiate only ED from both of the other groups (having no or slight stress symptomatology), was optimized by dichotomizing the replies to 26 items around the two highest scale steps (between “quite a bit” [3] and “very much” [4]), while the replies to 2 items were dichotomized in the midpoint of the scale (between the scale steps “somewhat”[2] and “quite a bit”[3]). This algorithm was based on the observation that the highest scale response (”much”) was seldom used by participants in the JS or the NJS+NLS groups, but relatively often by the ED respondents. A reply on step 4 was scored as “1” and a reply on step 3 or below was scored as “0.” After an equal weighting of the mean scores within each problem area/dimension A-F and multiplication of the mean of the means score (similar to the procedure for the SWS), a cutoff score of EWS=21.5 showed a detection rate of 86% of the former ED patients versus only 12% of the JS group and 2% of the NJS+NLS group, i.e., high sensitivity and specificity regarding detection of proper ED versus non-ED.

To summarize, the SWS seems to provide a suitable grading of stress symptomatology on a continuous scale from no appreciable signs to slight/moderate signs, as shown in the JS group. In cases where a person obtains a high SWS score, the EWS measure will indicate whether the stress symptomatology reaches an intensity that is also indicative of ED, or one that is more benign in nature.

***(C) Steps for calculating the SWS and EWS raw scores:***

*Calculation of the SWS scale score*

Step 1 - recoding of SWS items: All item scores 1 and 2 are recoded as 0 (zero), and all item scores of 3 and 4 are recoded as 1.

Step 2 - computation of SWS: SWS = 100 x [(Sum of scores on Item 1 to 3)/3) + ((Sum of scores of Item 4 to 7)/4) + ((Sum of scores of Item 8 and 9)/2) +((Sum of scores on Item 11, 12 and 14)/3) + ((Sum of scores Item 15 to 17)/3) + ((Sum of scores on Item 18 to 28)/11)] /6.

*Calculation of the EWS scale score*

Step 1 - recoding of EWS items: Items scores 1 to 3 are recoded as 0 (zero) and items scores of 4 are recoded as 1. On items 10 (control of work duties) and 13 (less able to make decisions), the scores 1 and 2 are recoded as 0 (zero) and items scores of 3 and 4 are recoded as 1.

Step 2 - computation of EWS: EWS = 100 x [(Sum of scores of Items 1 to 3)/3) + ((Sum of scores of Items 4 to 7)/4) + ((Sum of scores of Items 8 and 9)/2) +((Sum of scores on Items 10 to 14)/5) + ((Sum of scores Item 15 to 17)/3) + ((Sum of scores Item 18 to 28)/11)] /6.

Note that in cases of missing answers on any item in SWS or EWS, the divisor for that subgroup of items is reduced to reflect the mean value of the items that are responded to.

**Implementation on an experimental basis**

In 2009, LUCIE was compiled into a simple IT-based application, comprising an easy-to-use Excel sheet for computing and showing SWS and EWS in simplified diagrams, an extensive manual describing the proper use of LUCIE in clinical settings together with pertinent limitations of LUCIE, and graphics files with print-ready layouts for the LUCIE 28-item form. After preliminary testing, the first LUCIE package (version 1.0) was offered to occupational health services in Sweden through a personal license agreement in which each licensee agreed to provide feedback to the developers of LUCIE for further refinements of the contents and its user-friendliness. The ample clinical feedback provided by the licensees led to continuous improvements of all parts of the LUCIE package during the years that followed (versions 1.0a-1.0e; 1.1 and 1.2). In early 2014, LUCIE version 2.0 was made publicly available through the website [fhvmetodik.se](file:///C:\Users\ymed-rpn\LUCIE\Kai%20Lucie\Artikel%201\fhvmetodik.se), in the context of a larger project aiming to provide Swedish occupational health services with a wide range of evidence-based and easily administered and interpreted tools for clinical assessments. Patient data from clinical assessments have been collected and summarized by some of the users [[12](#_ENREF_12)] suggesting that LUCIE is a well-functioning clinical tool for identifying incipient ED. By time of LUCIE’s public release in early 2014, around 50 Swedish occupational health services had signed the license agreement for LUCIE.

**References.**

1. Karlson B, Jonsson P, Osterberg K: **Long-term stability of return to work after a workplace-oriented intervention for patients on sick leave for burnout**. *Bmc Public Health* 2014, **14**:821.

2. Karlson B, Jonsson P, Palsson B, Abjornsson G, Malmberg B, Larsson B, Osterberg K: **Return to work after a workplace-oriented intervention for patients on sick-leave for burnout--a prospective controlled study**. *Bmc Public Health* 2010, **10**:301.

3. Kecklund G, Åkerstedt T: **The psychometric properties of the Karolinska Sleep Questionnaire**. *J Sleep Res* 1992(6):221-229.

4. Maslach C, Jackson J, Leiter M: **Maslach Burnout Inventory Manual**. Palo Alto: Consulting Psychology Press; 1996.

5. Dallner M, Lindström K, Elo A, Skogstad A, Gamberale F, V H, Knardahl S, Örhede E: **Användarmanual för QPS-Nordic: Frågeformulär om psykologiska och sociala faktorer i arbetslivet utprovat i Danmark, Finland, Norge och Sverige.** In: *Arbetslivsrapport.* vol. 19. Stockholm; 2000.

6. Österberg K, Persson R, Karlson B, Eek F, Örbaek P: **Personality, mental distress, and subjective health complaints among persons with environmental annoyance**. *Human & Experimental Toxicology* 2007, **26**(3):231-241.

7. Netemeyer RG, Boles JS, McMurrian R: **Development and validation of work-family conflict and family-work conflict scales**. *J Appl Psychol* 1996, **81**(4):400-410.

8. Perski A: **Ur balans**. In*.* Stockholm: Bonniers 2002: 102-103.

9. Johnson JV, Hall EM: **Job Strain, Work Place Social Support, and Cardiovascular-Disease - a Cross-Sectional Study of a Random Sample of the Swedish Working Population**. *Am J Public Health* 1988, **78**(10):1336-1342.

10. Karasek RA: **Job Demands, Job Decision Latitude, and Mental Strain - Implications for Job Redesign**. *Administrative Science Quarterly* 1979, **24**(2):285-308.

11. Levi L, Bartley M, Marmot M, Karasek R, Theorell T, Siegrist J, Peter R, Belkic K, Savic C, Schnall P *et al*: **Stressors at the workplace: theoretical models**. *Occupational Medicine: State of the Art Reviews* 2000, **15**(1):69-106.

12. Tenenbaum A, Gram S, Ahlborg Jr G, Karlson B, Österberg K: **Implementation of a new screening method for incipient exhaustion disorder in occupational health services.** In: *International Congress on Occupational Health (ICOH)* Cancun, Mexico.; 2012.
