# Supplementary figures and images for: The Lund University Checklist for Incipient Exhaustion–a cross–sectional comparison of a new instrument with similar contemporary tools
Source: BMC Public Health. 2016 Apr 21;16:350. doi: 10.1186/s12889-016-3001-5 (PMC4839117; doi:10.1186/s12889-016-3001-5)

**Additional file 2. The LUCIE questionnaire, translated from the Swedish original**


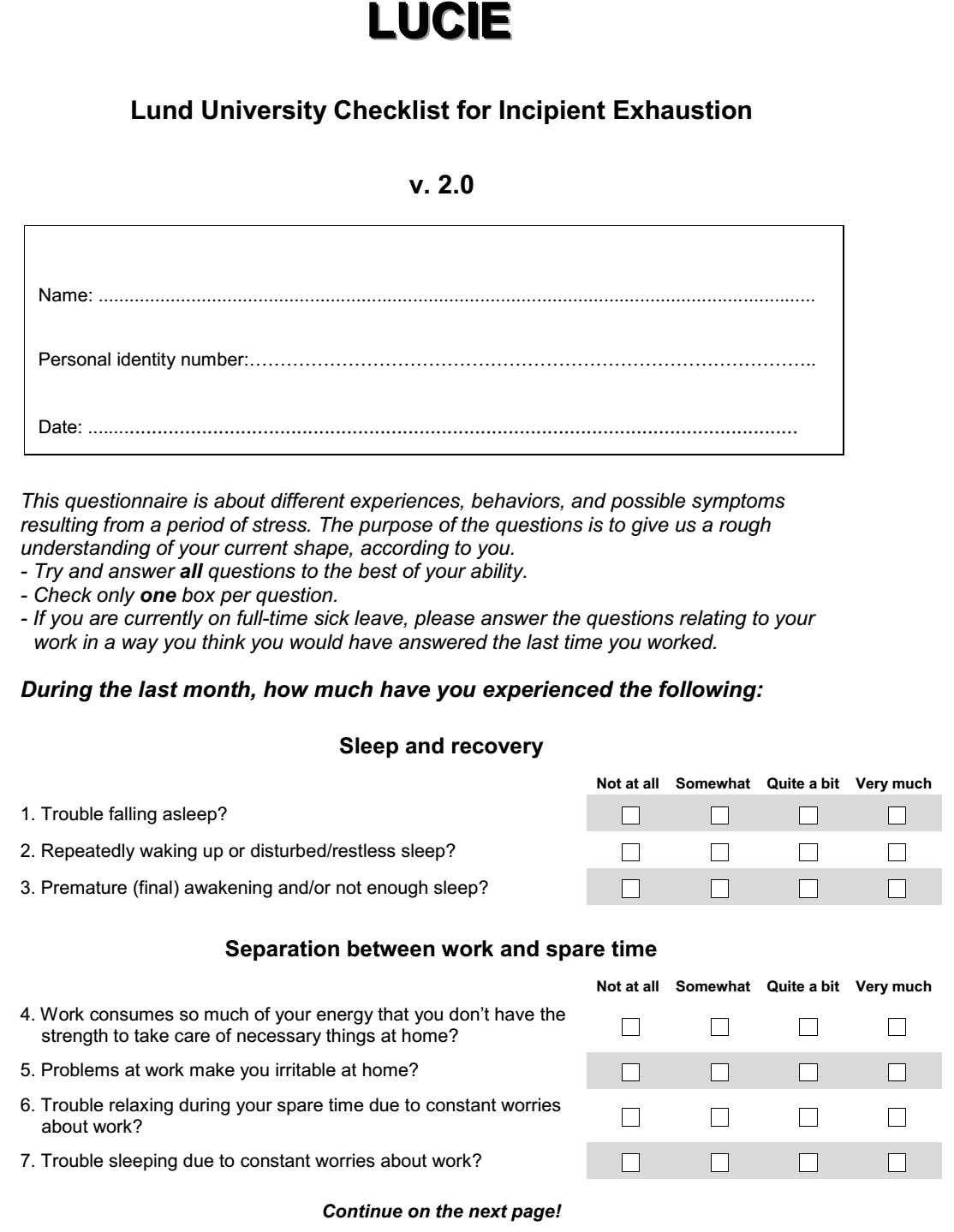


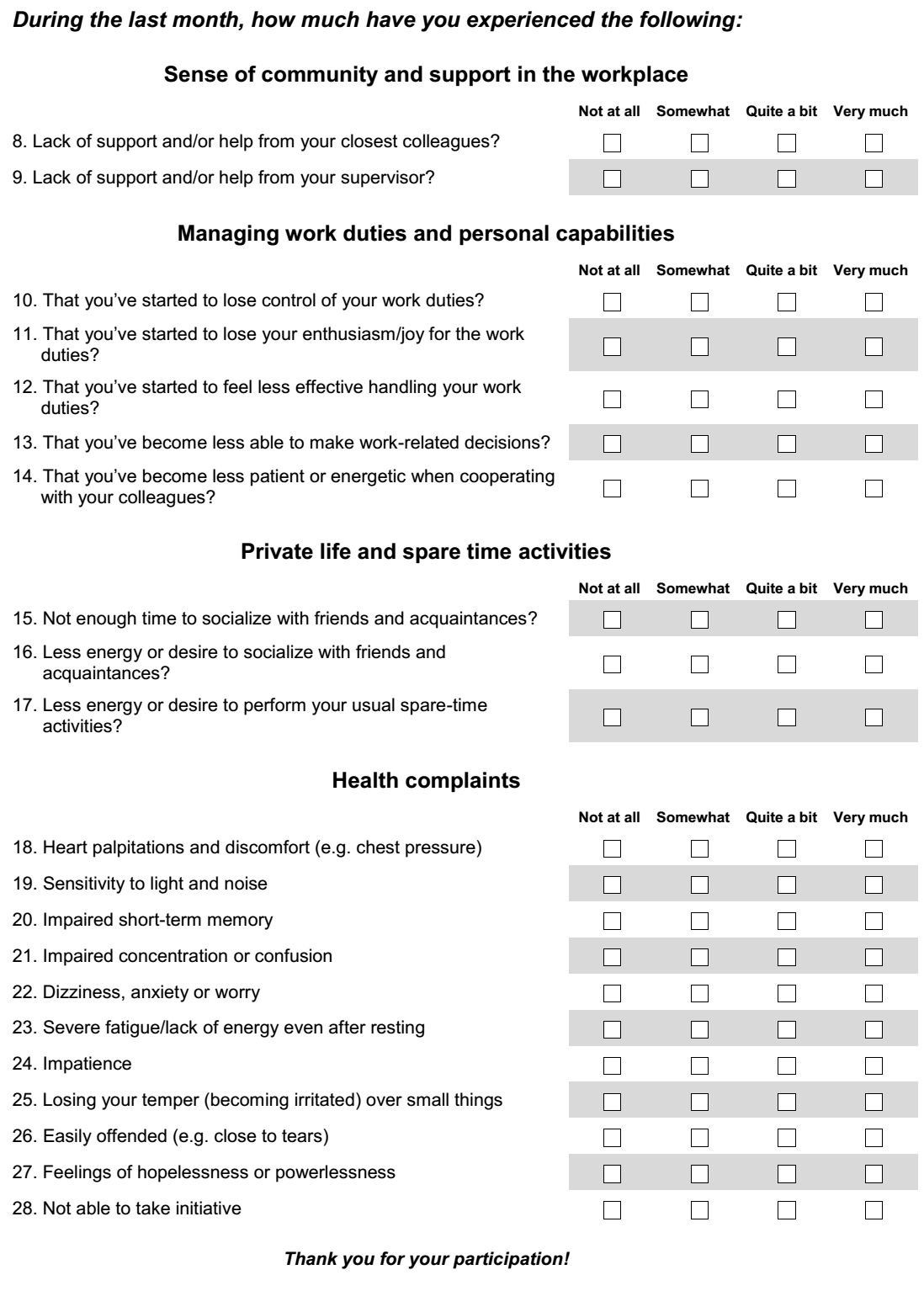

Supplement: Additional file 2: — The LUCIE questionnaire, translated from the Swedish original (DOCX 512 kb) [file 12889_2016_3001_MOESM2_ESM.docx]
